# Experience with etanercept, tocilizumab and interleukin-1 inhibitors in systemic onset juvenile idiopathic arthritis patients from the BIKER registry

**DOI:** 10.1186/s13075-017-1462-2

**Published:** 2017-11-22

**Authors:** Gerd Horneff, Anna Carina Schulz, Jens Klotsche, Anton Hospach, Kirsten Minden, Ivan Foeldvari, Ralf Trauzeddel, Gerd Ganser, Frank Weller-Heinemann, Johannes Perter Haas

**Affiliations:** 1 Asklepios Klinik Sankt Augustin, Sankt Augustin, Germany; 2 Charité Berlin, Klinik für Pädiatrie, Berlin, Germany; 3 Olgahospital Kinderklinik, Stuttgart, Germany; 4 Hamburger Zentrum für Kinder- und Jugendrheumatologie, Hamburg, Germany; 5 Helios Klinikum Berlin Buch, Klinik für Kinder- und Jugendmedizin, Berlin, Germany; 6 St. Josef-Stift, Sendenhorst, Germany; 7 Prof.-Hess-Kinderklinik, Bremen, Germany; 8 German Center for Pediatric and Adolescents´ Rheumatology, Garmisch-Partenkirchen, Germany

**Keywords:** Systemic onset juvenile idiopathic arthritis, Still’s disease, Anakinra, Canakinumab, Etanercept, Tocilizumab

## Abstract

**Background:**

Treatment of systemic onset juvenile idiopathic arthritis JIA (sJIA), although dramatically improved, remains a challenge. Experience from clinical practice will be presented using data from the German Biologics register (BiKeR) for evaluation of efficacy and safety of treatment with etanercept (ETA), tocilizumab (TOC) and the interleukin-1 inhibitors anakinra and canakinumab (IL-1i) in sJIA.

**Methods:**

Patients with sJIA documented in the BIKeR register, who were exposed to ETA, TOC or IL-1i were identified. Baseline demographics, clinical characteristics and disease activity parameters have been documented. Efficacy was determined using the JIA-American College of Rheumatology (ACR) response criteria and the Juvenile Disease Activity Score 10 (JADAS10). An intention-to-treat analysis was performed and patients who discontinued due to inefficacy or intolerance were analysed as non-responders. Safety assessments were based on adverse events (AEs) reports.

**Results:**

Since 2000, 245 sJIA patients (50.3% male) exposed to biologic agents have been identified: 143 patients treated with ETA, 71 with TOC and 60 with IL-1i (anakinra 38, canakinumab 22). All patients received systemic steroids for pre-treatment but less frequently with TOC and IL-1i than with ETA for concomitant treatment. At baseline, the ETA cohort had fewer systemic disease manifestations but more active joints. The JIA-ACR 30/50/70/90 response over a period of 24 months was reached more often in the IL-1i and TOC cohort than with ETA. ETA/TOC/IL1i JADAS-remission (JADAS ≤1) was reached in 20%/37%/52%, minimal disease activity (JADAS ≤3.8 in 35%/61%/68% and ACR inactive disease in 24%/33%/56%). As compared to ETA, rates of AEs were significantly higher in the TOC cohort (risk ratio (RR) 5.3/patient-year; *p* < 0.0001) and serious AE were observed more frequently with TOC (RR 2.5; *p* < 0.5) and IL1i (2.9; *p* < 0.01).

**Conclusions:**

A large proportion of patients gained significant response to treatment especially with TOC or IL-1is. After 6 months on treatment, JADAS remission was reached by up to half of patients while up to two thirds reached JADAS minimal disease activity. ETA has been used in the past but it is clearly less effective and its use in systemic JIA has markedly decreased in Germany.

**Electronic supplementary material:**

The online version of this article (doi:10.1186/s13075-017-1462-2) contains supplementary material, which is available to authorized users.

## Bullet points

- As compared to a comparator cohort treated with ETA a significantly higher number of patients with systemic JIA responded to treatment with TOC or the IL-1 inhibitors anakinra and canakinumab
- Improvement was observed in systemic symptoms as well as arthritis
- A significantly larger proportion of patients reached JADAS-remission upon treatment with TOC or upon IL-1is than with etanercept but only a minority of patients reached ACR preliminary criteria for inactive disease
- Serious adverse events including infections were more frequent upon treatment with TOC

## Background

Systemic juvenile idiopathic arthritis (sJIA) represents up to 10–20% of all JIA categories and is characterized by chronic arthritis, intermittently high, spiking temperatures up to 40 °C, maculopapular rash, hepatosplenomegaly, lymphadenopathy, serositis and a marked increase in the level of acute-phase reactants such as C-reactive protein (CRP) and erythrocyte sedimentation rate (ESR). The age at onset of disease is not limited to a specific period of childhood, but in a large cohort was found to accumulate within the first 6 years with a median age of disease onset of 4.7 years [1]. Girls and boys are affected in the same proportion [2]. Arthritis can be presented as mono-articular, oligo-articular or polyarticular predominantly affecting the cervical spine, hips, wrists and ankles but affecting smaller joints as well. One of the leading problems of sJIA is the potential joint destruction with luxation, ankyloses, and synostosis especially within polyarticular disease. Extra-articular manifestations are frequent with macrophage activation syndrome (MAS), a dreaded potentially life threatening complication still responsible for fatalities. Laboratory workup except, for elevated CRP and ESR, frequently shows distinct anaemia, leucocytosis and thrombocytosis, whereas mostly antibodies or rheumatic factors are not detected. At present, as the most severe JIA subtype, sJIA remains a challenge for prognosis and treatment.

Established therapies for treatment of JIA have been systemic glucocorticoids and nonsteroidal anti-inflammatory drugs (NSAIDs), which are associated with many side effects if given for years in this disease [3]. In contrast to their efficacy for treatment of arthritis, there is a poor clinical response to established JIA treatments such as methotrexate (MTX) or TNF-inhibitors of the systemic disease [4].

Studies have revealed that myeloid-related protein 8 (MRP-8 (S100A8)) and MRP-14 (S100A9), two calcium-binding S-100 proteins expressed and released by phagocytes, are highly elevated in active disease and may be used for diagnosis and for management of sJIA [5, 6]. The clinical characteristics of sJIA suggest that it is distinct from other forms of JIA, leading to the contention by some that sJIA should be separated from other forms of JIA and labelled as an autoinflammatory disease [7].

Thus, sJIA is regarded as an autoinflammatory disease due to the good response to inhibition of interleukin (IL)-1ß or IL-6 [8, 9]. Inhibition of the biologic activity of IL-6 by therapy with the monoclonal IL-6 receptor antibody tocilizumab (TOC) and inhibition of IL-1β with anti–IL-1 therapies such as canakinumab recently became approved by the US Food and Drug Administration (FDA) and the European Medicines Agency (EMA) for systemic JIA, and both have demonstrated remarkable benefit in randomized controlled trials [8–10]. In addition, anakinra, an IL-1 receptor antagonist has been used successfully in case series and in a randomized short-term pilot trial [11]. The improvement in therapeutic options prompted the Childhood Arthritis and Rheumatology Research Alliance (CARRA) to develop standardized consensus treatment plans for new-onset systemic JIA, including the use of biologic agents towards IL-1 or IL-6 [12]. In Germany, biologic therapy for JIA is monitored prospectively by the established Biologics register (BiKeR - Biologika in der Kinderrheumatologie). This enabled us to analyse the experience with several biologic treatments in patients with sJIA in clinical practice.

## Methods

The German BIKeR register has been approved by the local ethics committee of the Aerztekammer Nordrhein, Düsseldorf, Germany, reference number 2/2015/7441 [13]. Written consent was obtained from all patients and their parents and the data were collected pseudonymised. All data were collected prospectively including data on all patients in the German JIA-BIKeR registry, who had sJIA confirmed according to the International League of Associations of Rheumatology (ILAR) criteria, starting treatment with a biologic agent between 2000 and 2015. Patients were only included in analyses if they had assessments at baseline and at least at one follow up visit. Follow up visit reports were collected after 3 and 6 months and 6 monthly thereafter. Reminders were issued about missing and outstanding reports [14]. Patients’ characteristics included gender, age, diagnosis, disease duration, previous treatments and initial concomitant treatment and comorbidities. Clinical data included disease activity parameters, number of tender, swollen, active joints and number of joints with limitation of motion, physician´s assessment of global disease activity (visual analogue scale, VAS), patient’s/parent’s assessment of overall wellbeing and pain (both with the VAS), ESR, CRP and functional assessment according to the Childhood Health Assessment Questionnaire (CHAQ) disability index [15, 16].

### Effectiveness assessment

Effectiveness was determined using the JIA-American College of Rheumatology (ACR) response criteria and the Juvenile Disease Activity Score 10 (JADAS-10). The definition used for minimal disease activity was a JADAS-10 ≤ 3.8 and for remission on drug it was a JADAS-10 ≤ 1.0 [17, 18]. ACR preliminary criteria for remission and inactive disease (Wallace et al., [19]) were used including: (i) the lowest value of the physician’s judgement on global disease activity was 0 on a 100-mm visual analogue scale; (ii) ESR up to 20 mm/h; (iii) CRP up to 6 mg/l; (iv) morning stiffness lasting up to 15 min and (v) the absence of systemic manifestations (fever, rash, pericarditis, hepatomegaly, splenomegaly or lymph node swelling) to fulfil the definition of inactive disease/remission. The JIA-ACR 30/50/70/90 response described by the ACR is defined as 30%/50%/70%/90% or greater improvement in three or more of the six JIA core response variables without greater than 30% worsening in more than one of the remaining core response variables compared with baseline [20]. For this analysis, a 24-month treatment period was chosen as a meaningful time for primary judgement of early effectiveness and ongoing effectiveness and as a compromise with lower patient numbers with prolonged observation due to the nature of the registry analysis. In addition, the last available clinical status was used. The JADAS-10 is an established composite score for assessing disease activity including the four dimensions of physician’s global assessment of disease activity, patient’s global assessment of overall wellbeing, the active joint count and normalized ESR.

Effectiveness was analysed following the intention-to-treat principle and patients who discontinued due to inefficacy or intolerance were classified as non-responders. This study design is intending to avoid misleading artefacts.

### Safety assessment

Safety assessments included the collection of data on adverse events and serious adverse events. Adverse events were collected throughout the observation and specially requested on each routine follow up visit. In the case of adverse events the investigator assessed and recorded the adverse event in detail on the adverse event form including the date and time of onset, description, severity, time course, duration and outcome, relationship of the adverse event to the biological agent and alternative aetiology for events not considered “probably related” to the drugs that the patient received. Detailed data on adverse events of special interest were collected using special data forms.

### Statistics

The BiKeR data are entered into a Microsoft ACCESS™ (Microsoft Corp., Redmond WA, USA) database. Descriptive statistics were used to report the sociodemographic and clinical characteristics of the patients. This includes the absolute and relative frequencies of categorical data, and either the mean value with standard deviation or median with interquartile range for continuously distributed variables as appropriate. Comparisons of sociodemographic and clinical characteristics at baseline were conducted using the *t* test and chi square test. The patients cohort treated with etanercept (ETA) had been observed prospectively as well, although ETA had been approved earlier and was the only available biologic agent for treatment of patients with JIA for several years. Since more than 80% of the patients within this study had started treatment before 2008 the ETA cohort was used as a comparator group. In contrast, 74% started therapy after 2008 in the TOC and IL-1i cohort. No adjustments have been carried out for comparison of the ETA and the TOC/IL-1i cohorts, because of the remarkable differences between the cohorts in baseline characteristics, e.g. the concomitant conventional synthetic disease-modifying antirheumatic drug (csDMARD) and steroid use.

The propensity score was estimated for the comparison of effectiveness parameters between the TOC and IL-1 inhibitor cohorts. The likelihood of being treated by either TOC or an IL-1i was modelled by logistic regression including the predictor variables of year of treatment start, number of biologics in the history, concomitant csDMARD and steroid use, disease activity at treatment start, presence of systemic symptoms and disease duration at treatment start. All comparisons between the cohorts were adjusted by the propensity score in effectiveness analyses. Generalized linear mixed models were used to analyse the change in effectiveness parameters. Linear mixed models have the benefit that changes in parameters may be analyzed over time, (i) while using all study visits, (ii) to account for the dependence of measurements over time within the same patient and (iii) to model possible heterogeneity in the response between patients. The means of the linear mixed model were to be used for post-hoc tests to evaluate the change in each effectiveness parameter at a specified follow up. Comparison of adverse events and adverse event rates were calculated using the chi square and Wald test. Statistical analyses were conducted using STATA 12.1.

## Results

Within 245 patients with sJIA, 274 treatment approaches using biologics (ETA 143, TOC 71, anakinra 38, canakinumab 22) have been reported. Two patients on ETA had been exposed to a biologic before, compared to 48% in the TOC and 65% in the IL-1i cohort (Table 1). In total, the median age at start of treatment was comparable in all three cohorts (ETA (8.2); TOC (9.6); IL-1i (8.1)) as was the disease duration (3.3, 3.3, 2.8, years, respectively). However, the median disease duration differs between the TOC and IL-1i cohorts when stratifying by the number of previous biological DMARDS (bDMARDs) in the treatment history. Patients had median disease duration of 0.8 years (TOC 0.9, IL-1i 0.6) in first-line use, median disease duration of 5.8 years (TOC 7.4, IL-1i 5.2) in second-line use and median disease duration of 9.0 years in third-line use.

**Table 1:** Patient characteristics at baseline

	Etanercept	Tocilizumab 1. Biologic	Tocilizumab switcher	IL-1 inhibitors (anakinra or Canakinumab) 1. Biologic	IL-1 inhibitors (anakinra or canakinumab) switcher
Patient numbers	143	37	34	17	43 (30 + 13)
Gender (female)	72 (50.3%)	20 (54%)	17 (50%)	6 (35%)	22 (52%)
Age at onset (years)					
mean +/-SD	5.0 +/- 3.8	5.8 +/- 4.3	3.7 +/- 3.4	6.8 +/- 4.7	4.5 +/- 3.2
median (IQR)	4.1 (2.3; 6.4)	4.9 (2.2; 8.1)	2.6 (1.4; 4.4)	5.2 (3.5; 11.2)	3.7 (247; 5.3)
Age at bDMARD start (years)					
mean +/- SD	9.4 +/- 5.0	7.8 +/- 4.9	10.7 +/- 4.4	9.2 +/- 4.8	9.6 +/- 4.6
median (IQR)	8.2 (5.3; 13.0)	7.2 (3.2; 12.0)	10.6 (8.3; 13.7)	8.1 (5.1; 13.3)	8.4 (5.7; 13.2)
Disease duration (years)					
mean +/- SD	4.5 +/- 4.1	2.0 +/- 2.6	7.0 +/- 4.3	4.1 +/- 4.0	5.1 +/- 3.9
median (IQR)	3.3 (1.1; 6.6))	0.8 (0.3; 2.5)	7.4 (3.6; 9.8)	2.8 (0.6; 7.0)	5.2 (1.5; 8.7)
Pretreatment oral					
steroids	143 (100%)	37 (100%)	34 (100%)	11 (65%)	41 (95%)
steroid pulse therapy	24 (16.8%)	10 (27%)	6 (18%)	5 (29%)	12 (28%)
i.a. steroids	42 (29.4%)	4 (11%)	7 (21%)	0	10 (23%)
MTX	126 (88.1%)	29 (78%)	31 (91%)	8 (47%)	36 (83%)
other DMARDs	136 (95.1%)	6 (16%)	12 (35%)	3 (18%)	20 (47%)
biologics	2 (1.4%)	0	34 (100%)	0	39 (65.0%)
abatacept	0	0	1 (3%)	0	0
adalimumab	0	0	3 (9%)	0	3 (5.0%)
anakinra	2 (1.4%)	0	18 (53%)	0	n.a
canakinumab	0	0	2 (6%)	0	n.a.
etanercept	0	0	27 (79%)	0	32 (74.0%)
infliximab	0	0	1 (3%)	0	0
tocilizimab	0	0	0	0	9 (21%)
Concomitant treatment at enrolment					
steroids	118 (82.5%)	19 (51%)	12 (34%)	8 (47%)	19 (44%)
MTX	116 (81.1%)	25 (68%)	21 (62%)	5 (29%)	18 (42%)
other DMARDs	34 (23.8%)	3 (8%)	3 (9%)	2 (12%)	4 (10%)
Systemic manifestations at enrolment					
fever	1/13 (7.7%)	16/30 (53%)	7/32 (22%)	7/14 (50%)	5/16 (31%)
exanthema	2/13 (15.4%)	11/30 (37%)	7/32 (22%)	8/15 (53%)	2/16 (13%)
hepatomegaly	0/2 (0%)	9/20 (45%)	2/14 (14%)	1/9 (11%)	1/7 (14%)
splenomegaly	0/13 (0%)	7/30 (23%)	3/32 (9%)	2/15 (13%)	2/16 (13%)
serositis	0/13 (0%)	5/29 (17%)	1/31 (3%)	1/15 (7%)	1/16 (6%)
any systemic manifestation	2 (1.4%)	21/31 (68%)	9/34 (27%)	9/15 (60%)	7/16 (44%)
Disease activity joints					
swollen	114 (79.7%)	21 (57%)	21 (62%)	9 (56%)	19 (51%)
tender	116 (81.1%)	25 (68%)	21 (62%)	9 (56%)	21 (57%)
LOM	121 (84.6%)	25 (68%)	27 (79%)	8 (50%)	23 (62%)
active	120 (83.9%)	24 (65%)	25 (74%)	9 (56%)	22 (59%)
number of active joints mean +/-SD	9.0 +/- 11.2	5.1 +/- 8.5	5.6 +/- 8.4	2.3 +/- 2.3	3.2 +/- 4.8
median (IQR)	4.0 (2.0; 11.0)	3.0 (0.0; 6.0)	3.0 (0.3; 7.5)	2.5 (0.0; 4.3)	1.0 (0.0; 4.0)
ESR >20 mm/1 h	107/135 (79.3%)	21 (66%)	12 (55%)	4/8 (50%)	16/31 (52%)
CRP >6 mg/l	113/137 (82.5%)	26 (79%)	18 (64%)	11/15 (73%)	20/34 (59%)
JADAS-10 mean +/- SD (0–14)]	20.7 +/- 9.1	18.8 +/- 10.4	14.2 +/- 10.5	14.2 +/- 9.7	13.0 +/- 9.8
JADAS-10 CRP mean +/- (0–40)	22.2 +/- 9.8	22.3 +/- 11.2	15.3 +/- 9.9	4.3 +/- 10.2	13.4 +/- 10.6

*bDMARD* biological disease-modifying antirheumatic drug, *MTX* methotrexate, *ESR* erythrocyte sedimentation rate, *CRP* C-reactive protein, *JADAS* Juvenile Disease Activity Score, *LOM* limitation of motion

Pre-treatment consisted of systemic steroids in all patients starting biologics (Table 1). Initial concomitant treatment with systemic steroids was significantly less frequent in TOC-treated patients (44%, *p* < 0.001) and IL-1i-treated patients (45%, *p* < 0.001) compared to in the ETA cohort (83%). Concomitant MTX was used in the 83% of the TOC cohort, 58% of the IL-1i and in 88% of the ETA cohort.

Systemic manifestations, comprising fever, exanthema, hepatomegaly, splenomegaly or serositis, were present at baseline in 1.4% of patients treated with ETA, in 42.3% of patients treated with TOC and in 63% of patients treated with IL-1i. The mean numbers of active joints (+/- SD) were 9.0 +/- 11.2 with ETA, 5.4 +/- 8.4 with TOC and 2.9 +/- 4.2 with IL-1i. The mean baseline JADAS-10 (+/- SD) was highest in the ETA cohort (20.7 +/- 9.1), followed by the TOC cohort (16.2 +/- 10.6) and the IL-1i cohort (13.4 +/- 9.6).

### Effectiveness

Response to treatment with ETA, TOC or IL-1i was analysed separately for the presence of systemic symptoms, active arthritis, the JADAS-10 and remission defined as having a JADAS-10 score ≤1 and by the ACR definition of inactive disease.

The number/rate of patients without systemic symptoms increased with TOC (41/58%, 30/86%, 45/94%, 42/93%, 35/95%, 27/96%) and IL-1i (24/37%, 19/68%, 28/78%, 31/79%, 23/74%, 19/83%) at months 0, 3, 6, 12, 18 and 24 months, respectively.

The median (and IQR) of the active joint count and the number (rate) of patients with active joints are given in Table 2, showing a significant decrease in the number and rate of patients with active joints in all cohorts, while the rate of patients with active joints remained higher in the ETA cohort. Among patients with active joints at treatment start, patients treated by TOC had a higher likelihood of controlling the systemic symptoms compared to those treated with IL-1i (beta = 0.14, 95% CI, 0.03–0.60, *p* = 0.009; TOC, 87%, 97%, 90%, 100%, 95%; IL-1i, 65%, 71%, 77%, 58%, 71%, at months 3, 6, 12, 18, 24, respectively) on follow up. In detail, the difference in the presence of active symptoms between the two groups was significant at month 6 (beta = 0.10, 95% CI, 0.09–1.01, *p* = 0.051) and month 12 (beta = 0.08, 95% CI, 0.01–0.85, *p* = 0.036). There was no statistically significant difference between the two cohorts in the response for the control of systemic symptoms (beta = 1.22, 95% CI -0.46–2.91, *p* = 0.145) in patients without active joints at treatment start.

**Table 2:** Selected effectiveness parameters and steroid use

JADAS-10; median (IQR)^a^	Month 0	Month 3	Month 6	Month 12	Month 18	Month 24	Last observation
ETA	20.8 (14; 28.4)	6.9 (2.5; 14.3)	6.2 (1.1; 14.7)	3.8 (0.7; 15.7)	4.1 (1.5; 15.2)	3.3 (0.7; 9.4)	9.1 (2.1; 19.1)
TOC	16.9 (8.1; 24.8)	3.6 (0.8; 10.7)	1.5 (0.2; 3.8)	1.6 (0.4; 6.7)	0.9 (0.2; 2.0)	0.9 (0.1; 7.5)	0.9 (0.1; 4)
IL-1i	13 (6.7; 20.6)	0.8 (0.2; 1.6)	0.6 (0.2; 2.1)	0.8(0.2; 2.6)	0.2 (0; 1.9)	0.2 (0.1; 0.8)	0.8 (0.1; 5.1)
Patients with active systemic signs; *n* (%)^b^							
ETA	2 (1%)	0	0	2 (2.5%)	0	0	1 (1%)
TOC	30 (42%)	5 (14%)	3 (6%)	3 (7%)	2 (5%)	1 (4%)	4 (7%)
IL-1i	40 (63%)	9 (32%)	8 (22%)	8 (21%)	8 (26%)	4 (17%)	6 (11%)
Active joints; median (IQR)^a^							
ETA	4 (2; 11)	1 (0; 3)	1 (0; 5)	0 (0; 4)	0.5 (0; 4)	0.5 (0; 4)	1 (0; 6)
TOC	3.0 (0; 6)	0 (0; 0.5)	0 (0; 0.3)	0 (0; 1.0)	0 (0; 0)	0 (0; 1.0)	0 (0; 0)
IL-1i	2 (0; 5)	0 (0; 0.3)	0 (0; 0)	0 (0; 0)	0 (0; 0)	0 (0; 0)	0 (0; 0)
Patients with active joints; *n* (%)^b^							
ETA	121 (85%)	48 (53%)	49 (52%)	39 (48%)	36 (50%)	36 (50%)	67 (53%)
TOC	49 (69%)	9 (26%)	12 (25%)	13 (29%)	7 (19%)	8 (29%)	15 (21%)
IL-1i	34 (60%)	7 (25%)	6 (17%)	8 (21%)	7 (23%)	2 (9%)	12 (21%)
Patients on steroid use; *n* (%)^b^							
ETA	119 (83%)	66 (72%)	73 (76%)	48 (59%)	36 (49%)	35 (56%)	65 (49%)
TOC	32 (44%)	13 (35%)	16 (31%)	7 (14%)	6 (15%)	8 (27%)	7 (11%)
IL-1i	27 (45%)	12 (48%)	15 (45%)	10 (29%)	7 (25%)	6 (27%)	12 (24%)

Data as observed

*JADAS* Juvenile Disease Activity Score, *ETA* etanercept, *TOC* tocilizumab, *IL-li* interleukin-1 inhibitor

^a^Data are given as median and interquartile range of patients on treatment as observed. Note, the median value was higher in the last observation on ETA than on TOC or IL-1i

^b^Percentage of patients reported

At start of treatment, there were relatively more patients in the ETA cohort (121(85) with at least one active joint than in the TOC cohort (34/59.6%) or in the IL-1i cohort (49/69%). In the subgroup of patients with at least one active joint at treatment start, patients treated with TOC had a more pronounced decline in the active joint count as compared to those in the IL-1i cohort (beta = 0.19, 95% CI 0.06–0.33, *p* = 0.004) in follow up adjusted for the active joint count at baseline and the propensity score. A mean active joint count of 1.3 +/- 2.8 and 2.5 +/- 7.7 was reported for TOC and IL-1i (beta = -2.2, 95% CI, -4.1 to -0.4, *p* = 0.020) at 12 months of follow up and 0.8+/- 2.2 and 2.2 +/- 6.7 at 24 months.

Accompanying the improvement in clinical symptoms, a marked reduction in the use of systemic corticosteroids was observed (Table 2). Systemic steroids had been more frequently used in the ETA cohort than in the two others and a higher rate of patients had to remain on steroids despite biologic treatment.

A marked decrease in the mean JADAS-10 was noted in all three cohorts. The median (IQR) JADAS decreased in the ETA cohort from 20.8 (14.0–28.4) to 6.2 (1.1–14.7), in the TOC cohort from 16.9 (8.1–24.8) to 1.5 (0.2–3.8) and in the IL-1i cohort from 13.0 (6.7–20.6) to 0.6 (0.2–2.0) after 6 months of therapy with a further improvement with ongoing therapy (Table 2). At the last observation, the median JADAS-10 was lower in the TOC and the IL-1i cohorts than in the ETA cohort. The TOC and IL-1i cohorts did not significantly differ (beta = 0.12; 95% CI, -0.11 to 0.34; *p* = 0.321).

**Fig. 1:**
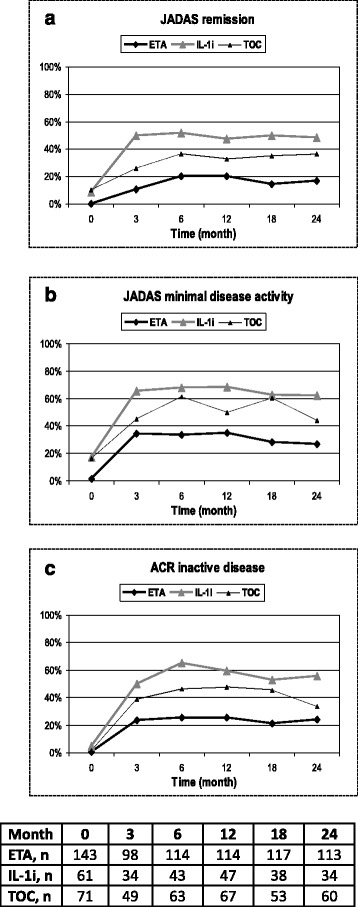
Juvenile Disease Activity Score 10 (JADAS-10) remission (defined as JADAS ≤1) and JADAS minimal disease activity (defined as JADAS ≤3.8). The number of patient contributing to the calculation is given below the figure. TOC, tocilizumab; ETA, etanercept; IL-1i, interleukin-1 inhibitor; ACR, American College of Rheumatology

Efficacy analysed in the intention-to-treat dataset is shown in Fig. 1. The intention-to-treat analysis revealed that JADAS-remission (JADAS-10 ≤ 1) was reached at month 3, 6, 12, 18 and 24 in 10/93, 21/105, 22/109, 16/110 and 18/109 patients in the ETA cohort, in 8/31, 16/44, 15/46, 15/43 and 15/41 in the TOC cohort and in 13/26, 16/31, 18/38, 16/32 and 14/29 in the IL-1i cohort, respectively. Minimal disease activity (MDA, JADAS-10 ≤ 3.8) was reached at month 3, 6, 12, 18 and 24 in 32, 35, 38, 31 and 29 patients in the ETA cohort, in 14, 27, 23, 26 and 18 patients in the TOC cohort and in 17, 21, 26, 20 and 18 patients in the IL-1i cohort, respectively (Fig. 1). At month 6, the difference between the rate of patients on treatment with IL-1i and TOC reaching JADAS-10 MDA compared to ETA was significant (ETA:TOC, *p* < 0.01; ETA:IL-1i, *p* < 0.001) as was the rate of patients reaching JADAS-10 remission (ETA:TOC, *p* < 0.05; ETA:IL-1i, *p* < 0.001). No significant differences were detected in JADAS-10 MDA in patients treated with TOC compared to patients treated with IL-1i (OR = 1.06; 95% CI, 0.96–1.16; *p* = 0.262) or in JADAS-10 remission (OR = 1.01; 95% CI, 0.94–1.09; *p* = 0.783) adjusted for the propensity score.

ACR-defined inactive disease was reached at month 3, 6, 12, 18 and 24 in 23/98, 29/114, 29/114, 25/117 and 27/113 in the ETA cohort, in 19/49, 29/63, 32/67, 24/53 and 20/60 in the TOC cohort and in 17/34, 28/43, 28/47, 20/38 and 19/34 in the IL-1i cohort, respectively.

The JIA-ACR responses, especially the high JIA-ACR70 and JIA-ACR90 were reached more often in the TOC and IL-1i cohort compared to the historical comparator ETA. A JIA-ACR70 response at month 3, 6, 12, 18 and 24 was only reached by 32%, 35%, 36%, 33%and 34% in the ETA cohort, by 44%, 46%, 40%, 44% and 40% in the TOC cohort and by 47%, 45%, 44%, 59% and 47% in the IL-1i cohort, respectively. The JIA-ACR90 response at month 3, 6, 12, 18 and 24 was reached by 16%, 19%, 22%, 18% and 19% in the ETA cohort, by 31%, 31%, 27%, 34% and 35% in the TOC cohort and by 34%, 36%, 35%, 51% and 41% in the IL-1i cohort, respectively (Additional file 1: Figure S1).

Discontinuations caused by inefficacy were significantly more frequent within the ETA cohort (43.4%) than within the TOC (8.5%) or IL-1i (21.7%) cohorts. However, TOC was more often discontinued due to intolerance compared to ETA or IL-1i (Table 3). No patients in the ETA cohort discontinued biologic therapy because of reaching remission whereas remission was reported to be the reason for discontinuation in 25.4% of the TOC and 18.3% of the IL-1i cohorts.

**Table 3:** Discontinuations (several reasons could be given in parallel

	Etanercept	Tocilizumab	Interleukin-1 inhibitor
Discontinuations	77 (53.8%)	28 (39.4%)	28 (46.7%)
Inefficacy	62 (43.4%)	6 (8.5%)	13 (21.7%)
Intolerance	5 (3.5%)	14 (19.7%)	1 (1.7)
Remission	0	18 (25.4%)	11 (18.3)
Others^a^	15 (10.5%)	3 (4.2%)	3 (5.0%)

^a^Other reasons: in the etanercept cohort, patient’s/parent’s wish (n = 6); no further visits by the patient (n = 2), treatment with approved biologic available (n = 2), no restart after an adverse event although it disappeared (n = 2), reason not reported (n = 3); in patients on anakinra, a patient was admitted to a clinical trial (n = 1), reason not reported (n = 2); in patients on tocilizumab, reason not reported (n = 3)

### Early effectiveness

Intention-to-treat analysis revealed a significant advantage of TOC and IL-1i over ETA (Fig. 1). At month 6, significantly more patients reached JADAS-remission (JADAS-10 ≤ 1) with TOC (36.4%; *p* = 0.05) or IL-1i (52%; *p* < 0.001) than with ETA (20%). Furthermore, significantly more patients reached a JADAS-MDA (JADAS-10 ≤ 3.8) with TOC (61%; *p* < 0.01) or with IL-1i (68%; *p* < 0.001) than with ETA (33%). There was no statistically significant difference between the TOC and IL-1i cohort in JADAS-remission (OR = 1.18; 95% CI, 0.39–3.57; p = 0.766) or in JADAS-MDA (OR = 1.73; 95% CI, 0.55–5.44; *p* = 0.349). ETA was more often discontinued due to inefficacy before month 6 (85.7%) than TOC (45.5%) or IL-1i (45.5%).

### Effectiveness in early disease

**Fig. 2:**
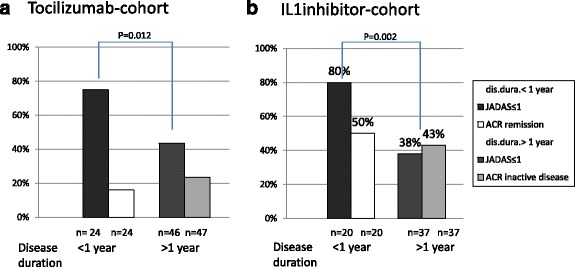
Juvenile Disease Activity Score (JADAS)-remission (JADAS-10 ≤ 1) and American College of Rheumatology (ACR) inactive disease (Wallace criteria) in patients in the tocilizumab cohort (**a**) and the interleukin-1 inhibitor (IL-1i) cohort (**b**) split according to disease duration <1 year and >1 year. At the last report in the early-treated cohort, 18 patients (75%) on tocilizumab and 16 (80%) on Il-1-inhibitors reached JADAS-remission significantly more frequently (OR 3.9 (95% CI, 1.3–11.6); *p* = 0.012 for tocilizumab and OR 6.6 (95% CI, 1.8–23.7; *p* = 0.002 for IL-1i) compared to 20 (44%) and 14 (38%), respectively. No difference was noted in the rate of patients reaching ACR inactive disease. Data are expressed as observed. The number of patients contributing to the calculation is given below the figure

There is a trend towards earlier treatment with biologics in sJIA. Therefore the efficacy of TOC and IL-1i in patients with sJIA who were treated within 12 months of diagnosis was compared to those with initiation of biologics later in the course of disease. There were 26 treatments (42%) started with TOC and 22 treatments started with IL-1i (36%) within the first 12 months of the disease. Earlier treatment led to a JADAS-10 ≤ 1 (JADAS-remission) at the last observation in 18 patients (75%) in the TOC cohort and 16 patients (80%) in the IL-Ii cohort. Fewer patients with sJIA who started later treatment with TOC or IL-1i reached JADAS remission (20 (44%) in the TOC cohort and 14 (38%) in the IL-1i cohort). These differences were significant for both TOC (OR 3.9 (95% CI, 1.3–11.6; *p* = 0.012) and IL-1i (OR 6.6 (95% CI, 1.8–23.7); *p* = 0.002) (Fig. 2a and b).

There was no correlation between disease duration and the rate of patients reaching ACR inactive disease. A total of 17 patients, 6 in the IL-1i cohort and 11 in the TOC cohort, who had reached JADAS remission, did not satisfy ACR inactive disease criteria due to a physician global assessment indicating values >0 (but below 10 mm on the 100 mm VAS) in 15 patients and a skin rash observed in 2 patients.

### Effectiveness in biologic-naïve patients compared to those pre-treated

**Fig. 3:**
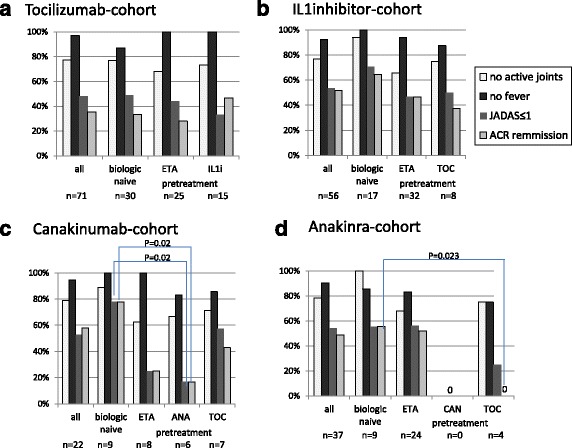
Last documented response for the tociizumab (TOC) cohort (**a**), combined IL-1 inhibitor (IL-1i) cohort (**b**), canakinumab cohort (**c**) and anakinra (ANA) cohort (**d**). The rate of patients with no active joints, no fever, Juvenile Disease Activity Score (JADAS)-remission, American College of Rheumatology (ACR)-defined inactive disease, ACR-remission is given, respectively, in the total cohort and for biologic-naive and pre-exposed patients. Differences in rates were calculated using the chi-square-test. Pearson’s *p* value is outlined if < 0.05. ETA, etanercept

Patients in the TOC cohort, the combined IL-1i cohort and the separate canakinumab and anakinra-cohort were sub-analysed by pre-treatment with ETA, IL-6 or IL-1i (Fig. 3). The reported number of patients with no active joints, no fever and either JADAS-remission (JADAS10 ≤ 1) and ACR inactive disease at the last report were used for comparison of biologic-naïve to pre-exposed patients. For the patients on TOC (Fig. 3a) and the total patients on IL-1i (Fig. 3b), comparison of remission rates (either ACR-defined inactive disease or JADAS-remission) assessed by the chi-square test there was no statistically significant difference between biologic-naïve and pretreated patients. Patients pretreated with anakinra significantly less frequently (*p* = 0.02) reached JADAS-remission and ACR-defined inactive disease upon treatment with canakinumab (Fig. 3c). There were only four patients who were switched from TOC to anakinra. None of them reached ACR-defined inactive disease on anakinra (Fig. 3d). Compared to the rate of 0.44 in biologic-naïve patients treated with anakinra, this observation was statistically significant (*p* = 0.02). No other significant influence of previous failure of biologic treatment was noted.

### Safety

There were 71 adverse events (AE) in the ETA cohort, 118 in the TOC and 81 in the IL-1i cohort (Table 4). Rates of AE (per patient-year) were significantly higher with TOC (risk ratio (RR) 5.3; *p* < 0.0001) compared to ETA and serious AE were also more frequent with TOC (RR 2.5; *p* = 0.01) and IL-1i (2.9; *p* < 0.01) compared to ETA. There were 11 reports of MAS, 3 occurring in the ETA cohort, 5 in the TOC and 3 in the IL-1i cohorts. Rates among the cohorts were not different. Of 110 infections, 11 were reported as serious. 3 were observed in the ETA cohort (2 patients with sepsis, one with gastroenteritis), 2 in the TOC cohort (1 patient with herpes zoster and 1 with pneumonia), 6 in the IL-1i cohort (1 patient with pneumonia, 1 with tonsillitis, 1 with enteritis and 1 with the common cold and 2 with bronchitis). Infection rates were significantly increased in both the TOC (RR 11.0; *p* < 0.00001) and IL-1i-cohorts (RR 7.4; *p* < 0.0001) compared to the ETA cohort. While serious infections were rare, rates were numerically higher in the IL-1i cohort. Each of one case of Hodgkin’s lymphoma, one of Crohn’s disease and one of demyelination occurred in the ETA cohort and one case of depression with suicidal thoughts occurred in the IL-1i cohort. There were two patients in the ETA cohort who died, both at the age of 16 years, due to septic shock and MAS. The events were considered as not related to biologics. One patient died 2 months after MAS was diagnosed and 6 months after discontinuation of ETA due to inefficacy. The other patient succumbed to septic shock and gastrointenstinal haemorrhage while on treatment with ETA. Before, the patient had been heavily pretreated with methotrexate, cyclophosphamide, chlorambucil and mycophenolate mofetil and corticosteroids. The large proportion of neurological signs and symptoms upon TOC were related to a single patient with complex seizure disorder with eight reports of seizures (three were classified as serious as the patient was admitted) but with onset of seizures before the start of TOC.

**Table 4:** Adverse event reports

	Etanercept		Tocilizumab			IL-1 inhibitors		
	*n* = 143; 355.8PY	Rate per patient year (95&CI)	*n* = 72; 111.6PY	Rate per patient year (95&CI)	Rate per patient year (95&CI)^a^	*n* = 60; 116.8PY	Rate per patient year (95&CI)	Rate per patient year (95&CI)^a^
AE	71	0.20 (0.16–0.25)	118	1.06 (0.88–1.27)	5.3 (3.9–7.1) *p* < 0.0001	81	0.69 (0.56–0.86)	3.5 (2.5–4.7) *p* > 0.05
SAE	18	0.05 (0.03–0.08)	14	0.13 (0.07–0.21)	2.5 (1.2–5.0) *p* = 0.01	17	0.15 (0.07–0.21)	2.9 (1.5–5.6) *P* = 0.002
JIA-Reactivation	4	0.01 (0.00–0.03)	7	0.06 (0.03–1.31)	5.6 (1.6–19.1) *p* = 0.006	10	0.86 (0.05–0.16)	7.6 (2.4–24.3) *p* = 0.0006
MAS	3	0.01 (0.00–0.03)	5	0.05/0.02–0.11)	5.3 (1.2–22.2) *p* = 0.02	3	0.03 (0.01–0.08)	3.0 (0.6–15.1) *p* > 0.05
hypersensitivity	5	0.01 (0.01–0.03)	4	0.04 (0.01–0.10)	2.6 (0.7–9.5) *p* > 0.05	2	0.02 (0.00–0.07)	1.2 (0.2–6.3) *p* > 0.05
infections	16	0.05 (0.03–0.08)	55	0.49 (0.38–0.64)	11.0 (6.3–19.1) *p* < 0.0001	39	0.33 (0.24–0.46)	7.4 (4.1–13.3) *p* < 0.0001
neutropenia	1	0.00 (0.00–0.02)	6	0.05 (0.02–0.12)	19.1 (2.3–158) *p* = 0.006	1	0.01 (0.00–0.06)	3.0 (0.2–48.7) *p* > 0.05
thrombocytopenia	1	0.00 (0.00–0.02)	1	0.01 (0.00–0.06)	3.2 (0.2–51) *p* > 0.05	1	0.01 (0.00–0.06)	3.0 (0.2–48.7) *p* > 0.05
elevated Transaminases	8	0.02 (0.01–0.45)	4	0.04 (0.01–0.10)	1.6 (0.5–5.3) *p* > 0.05	1	0.01 (0.00–0.06)	0.4 (0.05–3.0) *p* > 0.05
hair loss	4	0.01 (0.00–0.03)	2	0.02 (0.00–0.07)	1.6 (0.3–8.7) *p* > 0.05	0		
skin signs and symptoms	6	0.02 (0.01–0.03)	3	0.03 (0.01–0.08)	1.6 (0.4–6.4) *p* > 0.05	0		
neurol. signs and symptoms	3	0.01 (0.00–0.03)	11	0.10 (0.05–1.78)	11.7(3.3–42.0) *p* = 0.0001	0		

*AE* adverse event, *SAE* serious AE, *MAS* macrophage activation syndrome

^a^Wald test compared to the etanercept cohort

## Discussion

Severe, persistent systemic JIA still represents a major therapeutic challenge in pediatric rheumatology. We performed a systematic analysis of the German BIKeR register to evaluate and compare the effectiveness and safety of TOC, the IL-1i canakinumab and anakinra, and used ETA as a comparator. Both cohorts of patients receiving anakinra or canakinumab were pooled because of the small total numbers of patients receiving each medication compared to the other cohorts. No marked difference was observed between patients receiving one of these IL-1i.

Differences in baseline characteristics in regard to the ETA cohort as a comparator can be explained by the start of therapy at a different point of the pharmacological admission. Before the approval of ETA in 2000, no biological agent for the treatment of JIA was available. In contrast, 65% and 66%, respectively, of the cohorts of patients receiving TOC or one of the IL-1i were exposed to a biologic agent before. Patients in the IL-1i or TOC cohorts presented with more systemic disease activity, whereas active joints were the leading symptom in the ETA cohort, which makes direct comparison difficult. The low rate of active systemic features in the ETA cohort may be explained by the fact that ETA was approved in 2000 for treatment of polyarthritis in general. Patients with sJIA starting with systemic features during their course of the disease quite often have persistent articular disease with systemic features disappearing [21]. Thus, these patients were selected to receive a TNF inhibitor to treat articular manifestation of their disease. While a marked decrease in the mean JADAS was noted in all cohorts, the effectiveness of biologic treatment in patients with sJIA measured by the JADAS and JIA-ACR criteria resulted in higher response rates especially to IL-6 and IL-1i. The median JADAS decreased in the TOC cohort from 16.9 to 1.5 and in the IL-1i cohort from 13.0 to 0.6. ETA as the first biologic agent approved in the year 2000 for the treatment of JIA also led to a decrease of the JADAS from 20.8 to 6.2. The remarkable and significant difference in effectiveness between IL-1i and TOC compared to ETA were demonstrated in a larger proportion of patients reaching JADAS minimal disease activity or even JADAS remission under therapy.

Effectiveness in early disease upon either treatment with TOC or IL-1-inhibitiors was higher than in longer disease duration. This fits the observation that there has been a movement toward earlier treatment with biologics, probably because of a suggested “window of opportunity” that drives this trend, but still remains unproven. The influence of previous failure of a biologic was not pronounced. Failure to respond to ETA or an IL-1i seems not to predict response to TOC, whereas failure to respond to ETA or TOC has a numerical but not a statistically significant influence on the response to IL-1i. Only patients previously treated with anakinra had a markedly worse response to canakinumab, which is explained by their similar target but with a different mode of action. Since no patient switched from canakinumab to anakinra the opposite could not be analysed.

Adverse events (AE) and serious adverse events were significantly more frequent under therapy with TOC (RR 5.3, RR 2.5, respectively) and IL-inhibitors (RR 3.5., RR 2.9, respectively). The most frequently mentioned AE were infections. MAS was reported in all three cohorts. The occurrence of MAS with biologic therapy has been described before and biologics seem not to have a significant effect on risk of MAS or its clinical features in patients with systemic JIA [22]. Infections are the most common trigger, and MAS occurs even in patients whose systemic JIA is well-controlled with this treatment.

Neutropenia as a known side effect of TOC therapy was also observed in our population. In spite of the high rate of AEs under TOC or IL-1i, treatment discontinuation caused by intolerance was very rare within IL-1i (only one patient on canakinumab treatment who had MAS discontinued due to intolerance). However, biologic therapy was often discontinued due to remission of sJIA in the TOC and the IL-1i cohort.

Patients with JIA are already at greater risk of bacterial infections leading to hospitalization due to their chronic disease, the high disease burden and the high necessity for concomitant treatment with systemic corticosteroids. In summary, the safety profile regarding infections seems to be acceptable.

Previous clinical studies on TOC, canakinumab and anakinra demonstrated AE to occur more often in patients receiving a biological agent compared to their respective placebo group [8–11]. Especially, the rates of infections were reported to be higher in patients treated with TOC or canakinumab, as also seen in our analysis of data out of  the clinical  practice from the BIKeR register. Opportunistic infections were not reported although most of the patients had severe disease and were receiving combination therapies including biologics, immunosuppressants and moreover, corticosteroids. The pattern of AEs observed in this analysis of patients with sJIA is consistent with the known safety profile of biologics reported in other studies. They all were typical of those noted within other biological settings.

Clinical trials of TOC, canakinumab and anakinra in patients with sJIA have demonstrated the high efficacy of these biologic agents compared to placebos. These trials performed by De Benedetti et al. [9] and Yokota et al. [10] on the efficacy of TOC for treating sJIA show a large proportion of up to 90% of patients reaching JIA-ACR 30/50/70 response in week 6 and 12 of treatment. The same strength of efficacy was observed in two randomized clinical trials of canakinumab in patients with sJIA performed by Ruperto et al. [8] and in a clinical trial of anakinra [9]. For ETA no such trial has been performed for patients with sJIA, so a comparison with our results from the BiKeR register is not possible. Long-term observations of treatment with TOC, canakinumab and anakinra in patients with sJIA reveal similar results for efficacy assessment [22–26].

In terms of the efficacy and safety of the here mentioned biologic treatments for sJIA, the effectiveness and safety profile can be assigned as similar to the other JIA categories. In the CLIPPER study, ETA treatment was effective and well-tolerated in paediatric subjects with extended oligoarticular JIA, enthesites-related arthritis and psoriasis arthritis, with no unexpected safety findings [27]. In a long-term observation of patients with polyarticular JIA, ETA offers an acceptable safety profile and provides significant improvement in disease manifestations [28]. A phase 3, randomised, double-blind withdrawal trial of TOC treatment for polyarticular JIA resulted in significant improvement of signs and symptoms and TOC has a known safety profile reported in other phase 3 studies. The most common mentioned AEs were infections and neutropenia [29, 30]. The IL-1i canakinumab is not approved for JIA categories other than in systemic JIA, so a comparison of the efficacy and safety profile is not possible.

The comparison of effectiveness and safety between the ETA cohort and the IL-1i and TOC cohorts may be interpreted with caution and against the background of remarkably higher concomitant csDMARD and steroid use in the ETA cohort. It was not possible to adequately model this difference in a valid propensity score model; the differences remained after adjustments were made.

Beside this, there are a number of further limitations of our analysis. Data were gained from an observational study without any randomisation and a prolonged time period was necessary to gain larger numbers of patients. The decision to start and to stop treatment and the choice of the biologic was made by the responsible local physician with no predefined criteria. Nevertheless, this study provides the first indication for the comparison of different biologic agents in systemic JIA based on observational study data, with all their weaknesses, and demonstrates the need for well-controlled head-to-head studies for confirmation.

## Conclusions

The results revealed that a large proportion of patients with sJIA had a significant response to treatment, especially with TOC or with IL-1i. After 6 months on treatment, JADAS remission criteria were reached by up to 52% of patients while 61–68% reached JADAS minimal disease activity criteria. ETA has been used in the past. The rate of patients reaching remission is markedly lower than upon treatment with IL-1i or TOC. Its use in systemic JIA has markedly decreased in Germany. Moreover, it was used in patients presenting with arthritis with fewer systemic features [1] as suggested in the recommendation from the USA.

## Additional file

Additional file 1: Figure S1. Proportions of patients with a JIA-ACR 30/50/70/90 response from month 3 until month 24 as compared to baseline (week 0). The number of patient scontributing to the calculation is given below the figure. (PPT 1206 kb)

## Abbreviations

ACR: American Colleague of Rheumatology
AE: Adverse event
bDMARD: Biological disease-modifying drug
BIKER: Biologics in Pediatric Rheumatology Registry
CARRA: Childhood Arthritis and Rheumatology Research Alliance
CHAQ: Childhood Health Assessment Questionnaire
CRP: C-reactive protein
csDMARD: Conventional disease-modifying drug
DMARD: disease-modifying antirheumatic drug
EMA: European Medicines Agency
ESR: Erythrocyte sedimentation rate
ETA: Etanercept
FDA: Food and Drug Administration
IL: Interleukin
IL-1i: Interleukin-1 inhibitor
JADAS: Juvenile Disease Activity Score
JIA: Juvenile idiopathic arthritis
MAS: Macrophage activation syndrome
MDA: Minimal disease activity
MRP: Myeloid-related protein
MTX: Methotrexate
NSAIDs: Nonsteroidal anti-inflammatory drugs
OR: Odds ratio
PedACR: Pediatric ACR criteria
PsA: Psoriatic arthritis
RCT: Randomized controlled trials
RR: Risk ratio
SAE: Serious adverse event
SD: Standard deviation
sJIA: Systemic juvenile idiopathic arthritis
TNF: Tumour necrosis factor
TOC: Tocilizumab
VAS: Visual analogue scale
