# Supplementary figures and images for: Experience with etanercept, tocilizumab and interleukin-1 inhibitors in systemic onset juvenile idiopathic arthritis patients from the BIKER registry
Source: Arthritis Res Ther. 2017 Nov 22;19:256. doi: 10.1186/s13075-017-1462-2 (PMC5700562; doi:10.1186/s13075-017-1462-2)

## Slide 1
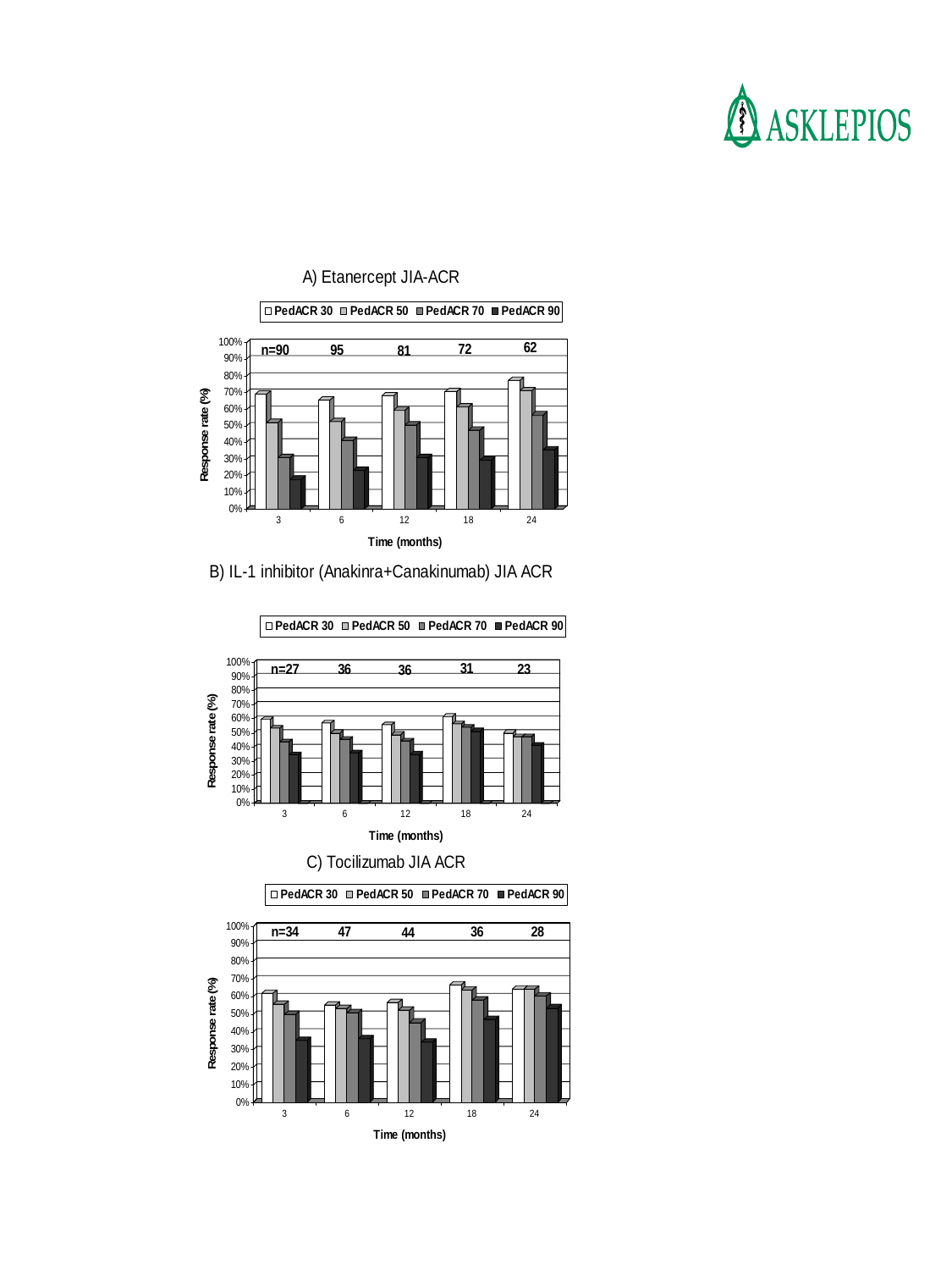

29.08.26
<number>

Supplement: Additional file 1: Figure S1. — Proportions of patients with a JIA-ACR 30/50/70/90 response from month 3 until month 24 as compared to baseline (week 0). The number of patient scontributing to the calculation is given below the figure. (PPT 1206 kb) [file 13075_2017_1462_MOESM1_ESM.ppt]
